# Can artificial intelligence optimize treatment planning and outcome prediction in fixed tooth- and implant-supported prosthodontics? A scoping review

**DOI:** 10.1186/s12903-025-07300-8

**Published:** 2025-12-11

**Authors:** Angkoon Khaohoen, Nobuhiro Yoda, Pimduen Rungsiyakull, Chaiy Rungsiyakull, Tenkumo Taichi

**Affiliations:** 1 Department of Restorative Dentistry, Faculty of Dentistry, Naresuan University, Phitsanulok, 65000 Thailand; 2 Division of Advanced Prosthetic Dentistry, Tohoku University Graduate School of Dentistry, Sendai, Miyagi 9808576 Japan; 3 Department of Prosthodontics, Faculty of Dentistry, Chiang Mai University, Chiang Mai, 50200 Thailand; 4 Department of Mechanical Engineering, Faculty of Engineering, Chiang Mai University, Chiang Mai, 50200 Thailand

**Keywords:** Artificial intelligence, Deep learning, Digital dentistry, Prosthodontics, Treatment planning, Outcome prediction, Clinical decision support

## Abstract

This review aims to explore the application of AI (artificial intelligence) in fixed prosthodontics and implant-supported fixed restorations, with a specific focus on the accuracy, effectiveness, and clinical applicability of AI models for optimizing treatment planning and predicting clinical outcomes. This review followed the PCC (Population, Concept, Context) framework. A systematic search was conducted using PubMed, Scopus, and Embase for studies published between January 2010 and July 2025. Keywords included “artificial intelligence,”, “deep learning”, “digital dentistry”, “prosthodontic treatment planning,” “clinical decision support,” and “outcome prediction.” The initial databases search yielded 834 results. After selection, 20 studies were included for analysis in this review. AI applications were grouped into four domains: implant planning, crown design, full-arch framework optimization, and prognostic modeling. Convolutional neural networks (CNNs), generative adversarial networks (GANs), regression models, and optimization algorithms were most frequently employed. In implant planning, AI achieved high accuracies (90–99.5%) for site detection, drilling protocols, and bone assessment. Crown design studies demonstrated occlusal and morphological deviations within clinically acceptable thresholds (0.18–0.30 mm) and mean internal gaps of 59–83 μm, while reducing design time by up to fourfold compared with conventional workflows. For full-arch prosthetics, optimization methods such as particle swarm optimization (PSO) and bi-evolutionary structural optimization (BESO) enhanced efficiency and reduced stress concentration in simulated workflows. Prognostic modeling showed promising performance, with models achieving over 90% accuracy in predicting implant survival and treatment outcomes. AI applications in prosthodontics are most developed in implant site planning and crown design, with fewer studies on full-arch optimization and prognosis. Models generally achieve high accuracy and efficiency, but most evidence is early-stage and simulation-based. Future research should focus on prospective validation, multimodal integration, and patient-centered outcomes to ensure clinical reliability.

**Supplementary Information:**

The online version contains supplementary material available at 10.1186/s12903-025-07300-8.

## Background

Artificial intelligence (AI) has demonstrated considerable promise across multiple domains of dentistry [1], including diagnosis, treatment planning, and outcome prediction. In diagnostic imaging, AI has been applied to caries detection [2], periodontal disease identification [3], and periapical lesion detection [4]. In orthodontics, AI supports diagnostic and treatment planning by enabling dentofacial image analysis, automated image classification, and enhanced accuracy with deep convolutional networks (DCNs) [5]. Al also contributes to outcome prediction, such as final tooth position and treatment duration, thereby improving efficiency, communication, and patient satisfaction [6].

In prosthodontics and implant dentistry, AI has been explored for restoration design, margin line detection, smile simulation, digital shade matching, and restoration monitoring [7, 8]. Several AI-modulated tools are now emerging that support clinical decision-making. For instance, generative adversarial networks (GANs) can automatically generate crown and bridge. In implantology, convolutional neural networks (CNNs) use cone beam computed tomography (CBCT) data to suggest implant drilling protocol and implant site. By integrating patient-specific data with three-dimensional (3D) models of the jaws and teeth, AI enhances accuracy, standardization, and efficiency in clinical workflows [8].

Despite these advances, most AI applications have focused on removable prosthodontics, as shown in a recent review highlighting progress in denture design automation, tooth arrangement, and component selection [9]. While implant planning and crown design have been the subject of several reviews, broader domains of fixed prosthodontics and implant-supported restorations, including full-arch optimization, and prognostic modeling, remain less investigated [10].

Although digital workflows have become widely adopted in prosthodontics, treatment planning for fixed restorations is still largely manual and dependent on individual clinical judgment, often leading to variability in decision-making and outcomes [10]. Existing AI applications in this field remain task-specific rather than integrative, such as models for implant fixture classification [11, 12] or AI-driven margin line detection [13]. However, the majority of these remain at the validation stage rather than being implemented in clinical practice. Far fewer studies have advanced toward prognostic modeling, outcome prediction, or comprehensive decision-support systems that integrate multimodal data, such as radiographs, CBCT, intraoral images, and patient-specific records [9, 10]. In implantology, there are promising efforts, for instance AI predicting implant site and outcome using CBCT data to support surgical planning [14].

This gap emphasizes the need for a comprehensive synthesis of current evidence. Unlike prior reviews focused narrowly on implant planning or crown design, this scoping review examines AI applications across the full spectrum of tooth- and implant-supported prosthodontics, including implant site planning, prosthesis design, framework optimization, and prognostic modeling. By emphasizing accuracy, effectiveness, validation strategies, and clinical applicability, this review aims to clarify the current landscape, highlight limitations, and provide a roadmap for future research.

## Methods

This scoping review was conducted to identify and synthesize studies on the application of AI in fixed prosthodontics and implant-supported restorations. The research question guiding the review was “How has AI been applied in fixed prosthodontics and implant-supported restorations, particularly in treatment planning, optimization, outcome prediction, and clinical decision support?”

### PCC (population, concept, context) framework

Following the PCC framework:

- Population (P):
  • Patients requiring fixed prosthodontics or implant-supported restorations.
  • Indirect populations represented through proxy data such as 3D-printed resin casts, partial arch scans, and foam block implant placements, which simulate clinical scenarios.
  • Datasets derived from academic or institutional sources (school datasets, case libraries, PubMed articles).
- Concept (C):
  • Application of AI for treatment planning, optimization, outcome prediction, or decision support.
  • AI models trained and validated on diverse input modalities such as intraoral scans, CBCT images, 3D-printed cast scans, and case datasets.
- Context (C): Clinical and laboratory workflows in fixed tooth- and implant-supported restorations.

### Search strategy

A systematic search was conducted to identify relevant publications from January 2010 through July 2025. The year 2010 was selected as the starting point because earlier applications of AI in dentistry were sparse and largely experimental, while significant growth in deep learning (DL) and medical AI began around this period. Three electronic databases (PubMed, Scopus, and Embase) were systematically searched using a combination of predefined keywords, including “artificial intelligence,” “deep learning”, “digital dentistry”, “fixed prosthodontics,” “treatment planning,” “optimization,” and “clinical decision support” [see Additional Table 1].

### Screening and selection

**Fig. 1:**
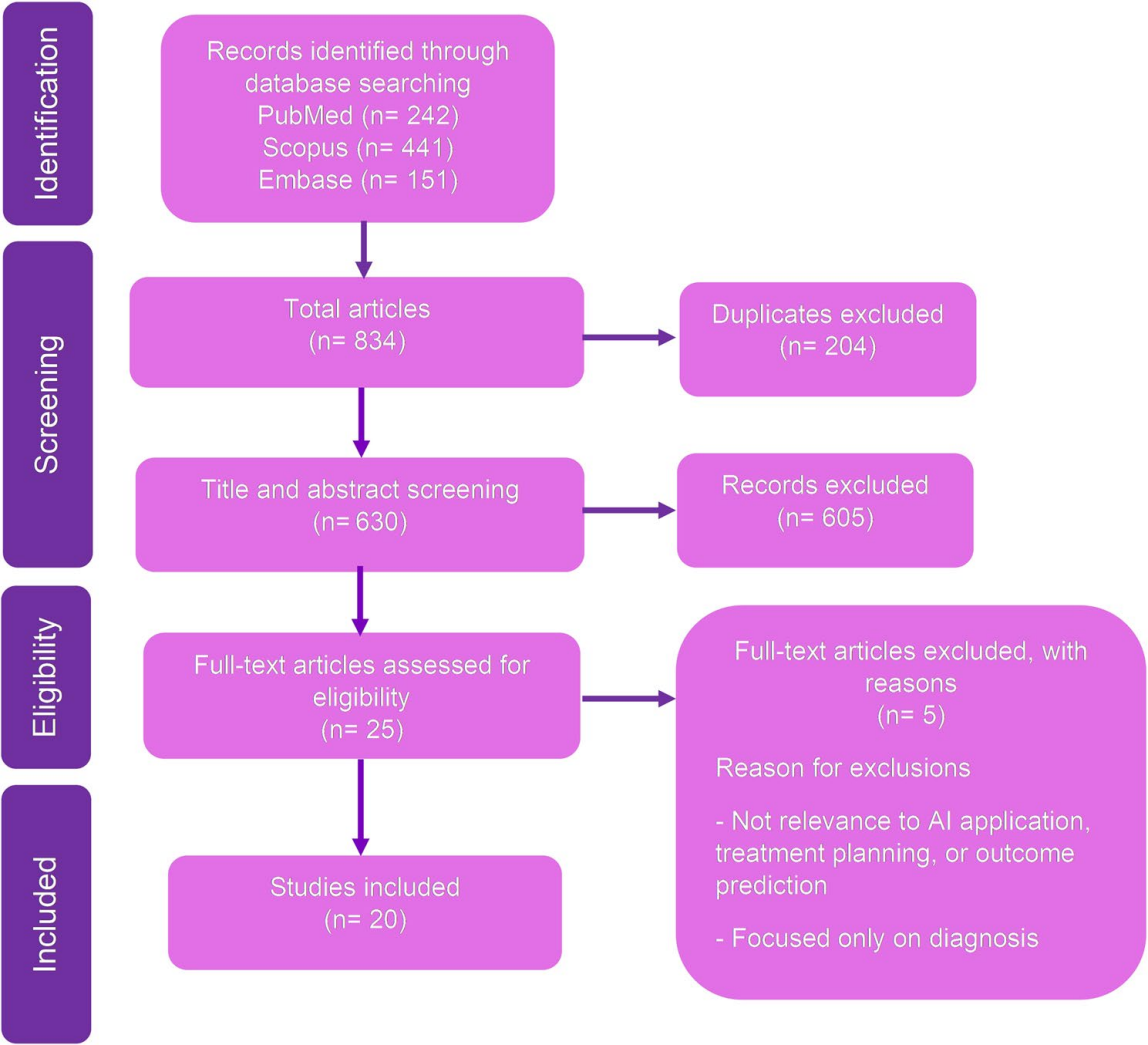
PRISMA flow diagram for the scoping review process

Inclusion and exclusion criteria were clearly defined prior to the search process (Table 1). All retrieved citations were imported into a software program (EndNote X9.3.3; Clarivate Analytics) for duplicate deletion, followed by a manual verification step. A two-phase screening process was carried out using another software program (Rayyan; Rayyan Systems, Inc). In the first phase, two independent reviewers (K.A., R.P.) performed a blinded screening of titles and abstracts based on the established eligibility criteria. In the second phase, 25 full-text articles were retrieved and assessed for inclusion. Any discrepancies during the selection process were resolved by consultation with a third reviewer (Y.N), an expert in the field of implant prosthodontics. At this stage, the reasons for exclusion were illustrated in Fig. 1.

**Table 1:** Inclusion and exclusion criteria for the search process

Inclusion criteria	Exclusion criteria
Clinical, in silico, and in vitro studies.	Literature review, editorials, commentaries, and non-peer-reviewed materials (except for relevant gray literature).
Studies focused on AI applications in fixed prosthodontics, including: - Tooth-supported prostheses (crowns and bridges) - Implant-supported fixed prostheses (single, multiple, or full-arch) - Fixed hybrid prostheses (All-on-4), where the restoration is non-removable by the patient.	Studies focused solely on removable prosthodontics (complete denture, partial denture, implant overdentures).
Focus areas may include treatment planning, decision support, or clinical outcome prediction, such as: - Biomechanical data (finite element analysis, occlusal load distribution, stress/strain patterns, and fatigue or fracture testing). - Clinical outcome data (prosthesis survival/failure, biological and technical. complications, patient-reported outcome, and radiographic or clinical follow-up results) - AI applications (prognostic modeling, treatment decision support, risk prediction for prosthesis failure or peri-implantitis).	Articles used AI only for diagnosis, without treatment planning or outcome prediction. Studies unrelated to prosthodontic (AI in orthodontics, periodontics, or other dental specialties).
Articles written in English that were published between January 2010 and July 2026.	Articles without the full text available.

Ultimately, 20 studies met all inclusion criteria and were included for analysis in this scoping review. The selection process is summarized in a PRISMA flow diagram (Fig. 1), which provides transparency in identification, screening, eligibility, and inclusion stages.

## Results

A total of 20 studies [15–34] were included in this scoping review, published between 2010 and 2025. These studies explored the application of AI across various stages of fixed- tooth and implant-supported prosthodontic treatment planning. The included methods spanned DL approaches (including CNNs), optimization algorithms, regression models, clustering method, and large language models (LLMs). Studies were categorized into four thematic groups such as implant planning and site prediction, crown design, full-arch and framework optimization, and prognostic modeling (Tables 2, 3, 4 and 5).

**Table 2:** Included studies based on implant planning and site prediction

Author(s), Year	AI model architecture	Purpose	Dataset source and size	Input data used	Treatment planning step involved	Performance metrics	Conclusion
Roongruangsilp and Khongkhunthian, 2021 [15]	Faster R-CNN with data augmentation	Object detection for planning implant position, size, and technique in posterior maxilla CBCT images	CBCT images from 184 patients (316 images)	Implant planning (size and technique) screenshots	Radiographic analysis and surgical planning preparation	Accuracy and detection improved with data size and varied by augmentation type	More training data and effective augmentation enhance AI accuracy for implant planning
Xiao et al., 2022 [16]	Nested-UNet	Segmentation model to classify bone density levels (types 1–5) from CBCT images	CBCT images from 70 patients (605 training sets)	CBCT images, HU-based jaw bone density maps	Bone quality assessment for drilling and implant site preparation	Dice score 0.75; HU prediction accuracy matched expert labels	This refined classification enables more accurate surgical planning by assisting clinicians in adjusting drilling parameters and selecting appropriate tools based on localized bone density during implant procedures
Alsomali et al., 2022 [17]	Mask R-CNN (with transfer learning)	Object detection for localizing GP markers in radiographic stents to mark proposed implant sites in CBCT	34 CBCT datasets with radiographic stents	Axial CBCT images with manually labeled GP markers	Initial implant site localization using radiographic markers	True positive rate: 83%, False positive rate: 2.8%, 17% missed	Demonstrated feasibility of AI-assisted marker localization; axial-only input limited model accuracy
Sakai et al., 2023 [18]	LeNet-5 (CNN-based model)	Predict the appropriate implant drilling protocol from CBCT images	1200 CBCT images from 60 patients	20 × 20 pixel regions from CBCT slices post-implant	Preoperative surgical protocol selection (drilling technique)	Accuracy: 93.7%, AUC: 98.6–99.4%	The AI model successfully predicted drilling protocols using compact CBCT image regions, showing promise as a decision support tool to improve primary stability.
Satapathy et al., 2024 [19]	DL (not specified)	Compare AI-generated and clinician-generated implant treatment plans	20 implant patient cases	CBCT-based planning parameters (position, angulation, depth)	Comprehensive implant planning (preoperative)	Mean deviation: position: 0.5 mm, angulation: 0.1°, depth: 0.1 mm; Planning time: AI: 10 min, clinical: 30 min	AI-generated plans closely matched clinical ones in positioning, angulation, and depth, while significantly reducing planning time, highlighting AI’s value in improving accuracy and efficiency.
Liu et al., 2025 [20]	MLR and DTR	Predict implant insertion torque based on anatomical and procedural factors to support immediate loading decisions	In vitro study; 324 implant placements in polyurethane blocks	Implant apex design, bone density, intraosseous depth, osteotomy protocol	Preoperative prediction of insertion torque	DTR model: R² = 0.951, RMSE = 3.648; MLR model: R² = 0.839, RMSE = 5.946	DTR model accurately predicted insertion torque using multiple input factors; provides a standardized tool for assessing immediate implant loading suitability.
Hashem et al., 2020 [21]	GLCTNN	Develop a RAIS to identify implant locations and improve surgical accuracy	PubMed articles, and Maryland schoolchildren dataset	Patient factors, bone and tissue info, dental caries, anatomical site features	Implant site detection and planning	Accuracy: 99.5%, Avg. deviation error: 0.323, Precision: 99.43%, Recall: 99.03%, F1-score: 99.3%, MCC: Highest among baseline models	The GLCTNN-based robotic system accurately identified implant sites and outperformed conventional ML methods, demonstrating strong potential for intelligent autonomous implant placement
Wu et al., 2025 [22]	LLM (GPT-4, Gemini, Claude, Qwen)	Compare the performance of multiple LLMs in clinical consensus, diagnosis, and treatment planning for implantology	20 simple questions, 20 complex questions, and 6 clinical cases from real implant scenarios	Questions and case data derived from ITI guidelines and hospital records	Diagnostic reasoning and treatment plan formulation	Accuracy: up to 80% (Gemini Pro, simple questions); Best overall: ChatGPT-4 in complex case handling; ICC: 0.965 (complex question), 0.797 (case analysis)	LLMs show promise as clinical decision-support tools, with ChatGPT-4 offering the most consistent responses; however, all models need refinement for complex, individualized planning

*AI* Artificial intelligent, *CBCT* Cone beam computed tomography, *CNN* Convolutional neural network, *DL* Deep learning, *DTR* Decision tree regression, *GLCTNN* Guided local search and continuous time neural network, *GP* Gutta percha, *HU* Hounsfield units, *ICC* Intraclass correlation coefficient, *ITI* International team for implantology, *LLM* Large language model, *MCC* Matthews correlation coefficient, *MLR* Multiple linear regression, *RAIS* Robotic-assisted intelligent system, *R-CNN* Regions with convolutional neural network, *RMSE* Root mean square error

**Table 3:** Included studies based on crown design and morphology optimization

Author(s), Year	AI model architecture	Dataset source and size	Input data used	Treatment planning step involved	Performance metrics	Conclusion
Cho et al., 2023 [23]	GAN (StyleGAN, Pixel2Style2Pixel)	30 clinical cases with posterior teeth	Intraoral and cast scans	Crown design optimization	Better time efficiency, morphology fit, internal fit (*p* < 0.001)	AI software improves crown design outcomes and efficiency
Cho et al., 2024 [24]	GAN (StyleGAN), CNN	30 partial arch scans (AI and manual compared)	STL files from digital impressions	Crown design (morphology, occlusion, fit)	Comparable or superior fit, morphology deviations ~ within ± 50 μm	DL systems offer clinically acceptable outcomes; reduce technician workload
Cho et al., 2024 [25]	CNN GAN (StyleGAN, Pixel2Style2Pixel)	20 3D-printed resin casts (posterior ISCs)	STL scans, implant scan bodies, digital impressions	Design of ISC (contour, emergence, occlusion)	Time efficiency high, morphology comparable, occlusal contact *p* < 0.004	AI-generated ISC shows clinical feasibility and reduces workload with comparable or better precision
Jin et al., 2023 [26]	CSM, Biomechanical Simulation	Library of oral scans; size not specified	3D scans of adjacent teeth and preparation	Crown design and biomechanics-based optimization	Biomechanical simulation; less stress concentration	Fully automated crown design shows high anatomical and biomechanical fidelity
Chau et al., 2024 [27]	3D-GAN	169 participants = 159 training casts and 10 validation casts	Digitized casts (original vs. processed, maxillary right first molar removed)	Automated design of single-molar dental prosthesis	Mean Hausdorff distance 0.633 ± 0.961 mm; IoU = 0.600 (60%)	Feasible AI design of biomimetic single-molar prostheses; accuracy acceptable but needs further optimization
Gu et al., 2023 [28]	Conditional GAN	400 pairs of tooth images (300 training, 100 test)	Mask image of missing tooth, opposing tooth, target pits/fissures	Reconstruction of functional occlusal pits and fissures in crowns	Standard deviation 0.1802 mm versus expert models; PSNR up to 23.38, SSIM up to 0.8412	Restores detailed pits/fissures effectively; improves crown morphology with accurate occlusion features

*3D* Three-dimensional, *AI* Artificial intelligence, *CNN* Convolutional neural network, *CSM* Conditional shape model, *DL* Deep learning, *GAN* Generative adversarial networks, *IoU* Intersection over union, *ISC* Implant supported crown, *PSNR* Peak signal-to-noise ratio, *SSIM* Structural similarity index measure, *STL* Stereolithography

**Table 4:** Included studies based on full-arch and framework optimization

Author(s), Year	AI model architecture	Dataset source and size	Input data used	Treatment planning step involved	Performance metrics	Conclusion
Chen et al., 2024 [29]	ANN and PSO	2800 FEA simulations	Implant parameters and mandible geometry (13 variables)	Optimized implant configuration planning	11.08% stress reduction; accuracy within 10.4% avg. error	Rapid, accurate configuration suggestion for each patient (30s), enabling chairside optimization
Chen et al., 2024 [30]	CNN (Inception V3) with BESO optimization	14,994 BESO-FEA training sets	Implant position, angulation, jaw shape, loading	Framework structural optimization	Compliance error 0.29%, shape error 11.26%	Significant reduction in computing time with AI (6.5 h → 45 s), promising for real-time clinical structural planning

*AI* Artificial intelligence, *ANN* Artificial neural network, *BESO* Bi-evolutionary structural optimization, *CNN* Convolutional neural network, *FEA* Finite element analysis, *PSO* Particle swarm optimization

**Table 5:** Included study based on prognostic and outcome prediction tools

Author(s), Year	AI model architecture	Dataset source and size	Input data used	Treatment planning step involved	Performance metrics	Conclusion
Lyakhov et al., 2022 [31]	ANN (Multilayer Perceptron)	1646 patient case records, 112 statistical variables	Patient demographics, habits, comorbidities, implant factors	Predicting implant survival	Prediction accuracy = 94.48%	Promising prognostic tool; not approved for autonomous clinical use
Liu et al., 2024 [32]	Interpretable MIL with Hosmer–Lemeshow–based loss function	1,627 patient cases	Clinical and implant-related features.	Risk prediction of implant failure	p-value, MSE, Hamming-Loss, and clinical risk stratification tests	MIL model provided better-calibrated, interpretable predictions; identified key predictors; supports personalized implant treatment planning
Xie et al., 2024 [33]	Hybrid Unsupervised Clustering (Hierarchical + k-means)	8,513 patient cases	Age, blood glucose, smoking history, implant diameter, implant length, implant position, bone quality, and other clinical/implant features	Risk prediction for implant survival	Clustering validity indices (e.g., Silhouette score, Dunn index, Davies-Bouldin index)	Hybrid model improved clustering quality, stratified patient risk more clearly, and supports personalized treatment planning.
Zhu et al., 2025 [34]	AMDRN	CBCT imaging dataset of sinus floor elevation implant patients	Anatomical CT imaging features, radiomics signatures, clinical parameters	Predicting implant failure risk in sinus floor elevation cases	Prediction accuracy = 90%, AUC = 0.93	AMDRN improved prediction accuracy, integrating anatomical and clinical data; supports precise preoperative risk assessment and better treatment planning

*AMDRN* Anatomically based multitask deep learning radiomics nomogram, *ANN* Artificial neural network, *AUC* Area under the curve, *CNN* Convolutional neural network, *MIL* Multi-instance learning, *MSE* Mean squared error, *SVM* Support vector machine

## Implant site analysis and treatment planning

Eight studies [15–22] applied AI for preoperative implant planning. Two studies [15, 17] focused on object detection, one model [16] addressed bone density classification, two studies [19, 22] compared AI versus dentist-generated treatment plans, two studies [18, 20] developed models for implant drill and torque prediction, and one study [21] investigated implant location. Three studies [15–17] focused on detection and segmentation tasks, one study [21] focused on optimization model, and four [18–20, 22] developed prediction models. Five studies [15–19] used 2D/3D CBCT images as training data sources, while one study [22] used text-based inputs through LLMs. One study [21] used real patient data, one study [20] was based on in vitro stimulation. CNN models were applied in three studies [15, 17, 18], while other studies used Nested U-Net [16], decision tree regression (DTR) [20], guided local search and continuous time neural networks (GLCTNN) [21] and LLMs [22], while one study [19] did not specify the model. LLMs differ from image-based programs, as they process textual data such as clinical guidelines and literature rather than radiographic imaging. Five studies [15, 17, 18, 20, 21] used pre-specified validation datasets to measure the performance metrics and three studies [16, 19, 22] validated in real clinical scenarios with clinicians.

Object detection studies could accurately localize radiographic markers and treatment landmarks. Alsomali et al. developed a model that detected stent markers in 34 CBCT images with an 83% true positive detection rate and 2.8% false positive rate [17], Similarly, Roongruangsilp et al. demonstrated the learning curve of Faster R-CNN models trained on 316 CBCT implant position images, with detection rates improving as the training dataset increased, and data augmentation strategies (e.g., sharpening and coloring) enhancing detection by up to 40% in panoramic images and 18.59% in cross-sectional images [15]. The bone density classification models were also developed to support implant planning. Xiao et al. construct a Nested U-Net-based model to classify jaw bone mineral density, achieving a dice score of 0.75 on 605 CBCT training sets, particularly across 70 mandibular jaws [16]. Prediction models extended AI into intraoperative guidance. Sakai et al. proposed a LeNet-5 model trained on 1,200 CBCT slices, which predicted implant drilling protocols with 93.7% accuracy [18]. Liu et al. further developed prediction models for insertion torque in 324 immediate implants tested in polyurethane blocks, with The DTR model achieving an R^2^ of 0.951 [20]. AI-generated treatment planning was also compared against clinician expertise. Satapathy et al. reported that, in 20 patients. AI-generated plans achieved discrepancies of less than 1 mm in position and less than 2 degrees in angulation compared with clinical plans, while reducing planning times from 30 min (clinicians) to 10 min (AI) [19]. Innovative approaches such as robotics and LLMs further expanded the application domain. Hashem et al. integrated GLCTNN into robotic implant systems using PubMed and Maryland school children datasets, achieving 99.5% accuracy in determining implant locations with an average deviation error of 0.323 [21]. Meanwhile, Wu et al. benchmarked LLMs (ChatGPT-4, Gemini, Claude, Qwen) on dental implant consensus tasks (20 simple questions, 20 complex questions, and 6 clinical scenarios). Gemini achieved the highest accuracy rate (0.80) in simple tasks, while ChatGPT-4 demonstrated the most consistent performance, with the highest mean score (7.99 ± 1.95) in complex case handling [22].

### AI-assisted crown design

Six studies [23–28] applied AI for preoperative crown design and evaluation. All studies focused on automating the design of single crowns or implant-supported prostheses, primarily for posterior teeth. Most models were trained on 3D intraoral or CBCT-derived datasets [23–27], while one study specifically addressed occlusal surface image generation using tooth depth images [28]. GANs were applied in five studies [23–25, 27, 28], while one study used conditional shape model (CSM) [26].

Object generation tasks using GANs demonstrated promising accuracy in crown morphology reconstruction. Cho et al. developed a model that designed crowns for 30 posterior abutments, achieving shorter design times, improved internal fit, and reduced occlusal deviation compared with conventional CAD systems (*P* < 0.05) [23]. Similarly, Cho et al. compared DL-based crowns (AA and AD groups) with technician-designed crowns (NC group) in 30 partial arch scans. AI-generated crowns showed occlusal surface morphology deviations of 251.8–266.3 μm and axial surface deviations of 185.9–231.7 μm. Mean internal gaps were 83.1 ± 13.1 μm in the AA group, 59.4 ± 12.1 μm in the AD group, and 65.4 ± 10.3 μm in the NC group, with the AD group exhibiting the best internal fit with the prepared abutment. In terms of cusp angles, all of groups fell within the optimal range (50° to 70°). All parameters remained within clinically acceptable thresholds [24]. For implant-supported restorations, Cho et al. evaluated crowns using 20 3D-printed resin casts of partially edentulous arches. Their study demonstrated that DL-designed crowns (DB and DM groups) achieved comparable outcomes to technician designs (NC group) in occlusal table area, cusp angle, cusp height, proximal contact, and emergence profile (all *P* >0.05), with only slight differences in occlusal intensity. However, DL-based crowns showed significantly shorter working times (83.9 ± 30.4s) approximately four times faster than non-DL-based crown (370.3 ± 98.3s) [25].

Beyond conventional morphology replication, Jin et al. developed an anatomically driven AI system trained on 20 intraoral scans (10 maxillary and 10 mandibular first molars), which optimized both crown morphology and biomechanical properties. The AI-generated crowns achieved Chamfer Distance errors of 0.26 ± 0.08 mm for maxillary first molars and 0.28 ± 0.07 mm for mandibular first molars, well within the 0.5 mm clinical threshold. The optimized porous internal structures demonstrated ~ 70% porosity with 0.2–0.6 mm wall thickness, resulting in more uniform stress distribution compared with solid crowns, particularly reducing stress concentrations at cusp regions [26].

Chau et al. conducted a feasibility study on AI-designed single-molar prostheses using 169 digitized casts (159 for training and 10 for validation) matched to natural molar teeth. Their 3D GAN-based system produced crowns with a mean Hausdorff distance of 0.441–0.752 mm (average 0.633 mm) and an Intersection-over-Union of 0.600 (60%), confirming that AI-generated molars could replicate natural tooth morphology with clinically acceptable fidelity [27]. Gu et al. proposed an image generation technology for functional occlusal pits and fissures using a conditional GAN trained on 400 pairs of tooth images, which restored detailed crown surface morphology. The generated crowns achieved a mean deviation of only 0.180 mm compared with expert-designed crowns, significantly improving the reproduction of fine occlusal anatomy and surpassing template-based CAD methods in functional surface accuracy [28].

### Full-arch prosthetics and framework optimization

Two studies [29, 30] applied AI for full-arch prosthetics and framework optimization. One study [29] optimized implant design and placement within the complete-arch context, while the other [30] focused on structural optimization of the prosthetic framework. Both studies relied on finite element analysis (FEA)-derived datasets, integrated with AI models to accelerate computation and guide biomechanical planning [29, 30]. One study [29] employed an ANN combined with particle swarm optimization (PSO) [29], whereas another study [30] used a CNN embedded in a bidirectional evolutionary structural optimization (BESO) framework [30].

Chen et al. introduced ANN-PSO trained on 2,800 FEA simulations to optimize implant design in full-arch prosthetics. When verified through FEA, the model achieved an average prediction error of 10.4 ± 8.1%. Compared with conventional FEA–PSO methods, computation time was reduced from 3 days to < 60 s. Biomechanical validation further showed that ANN–PSO reduced peri-implant stresses by an average of 11.08% across test cases [29]. Chen et al. developed BESO-Net, a CNN-based bidirectional evolutionary structural optimization system for All-on-4^®^ prosthetic frameworks. Using 14,994 FEA-derived configurations, the model achieved a compliance prediction error of only 0.29% and shape error of 11.26% compared with traditional BESO-FEA methods. Importantly, optimization time was reduced from 6.5 h to 45 s [30].

### Prognostic modeling

Four studies [31–34] applied AI for prognostic modeling of implant survival and failure risk. Three studies [31–33] developed prediction models using large-scale clinical datasets, while one [34] integrated imaging-derived radiomics or multi-modal features for individualized risk assessment. Model architectures varied across studies, including a multilayer perceptron neural network [31], an interpretable multi-instance learning (MIL) framework [32], a hybrid unsupervised clustering model [33], and an anatomically based multitask deep learning radiomics nomogram (AMDRN) [34].

Lyakhov et al. developed a multilayer perceptron neural network trained on 1,646 patient cases with 112 clinical factors. Their system achieved a recognition accuracy of 94.48% for predicting single-implant survival [31]. Zhu et al. proposed the AMDRN framework combining 3D CBCT-derived radiomics, anatomical segmentation, and clinical variables for sinus floor elevation cases. The AMDRN achieved 90% accuracy and an AUC of 0.93, outperforming both radiomics-only and clinical-only nomograms, and enabling individualized risk assessment [34].

Liu et al. introduced an interpretable MIL framework trained on 1,627 patient datasets collected over 23 years, integrating 55 clinical and biological features. By integrating a Hosmer–Lemeshow–based calibration loss, the model highlighted bone density as the most influential predictor. It provided stable risk stratification and transparent decision pathways, enhancing clinical interpretability [32]. Xie et al. applied a hybrid unsupervised clustering method combining two-step hierarchical clustering and k-means with Cox regression and Kaplan–Meier survival analysis on 8,513 implants. Six clusters with distinct prognoses were identified. Key high-risk factors included age, smoking history, implant diameter, implant length, implant position, and surgical procedure [33].

## Discussion

This scoping review highlights that AI applications in fixed tooth- and implant-supported prosthodontics are most concentrated in the preoperative phase, particularly in implant site planning and crown design, with fewer studies exploring full-arch framework optimization or long-term outcome prediction. Across these processes, AI was primarily used to support implant detection and planning [15–22], automate crown morphology and fit [23–28], optimize biomechanical frameworks [29, 30], and predict implant survival [31–34]. Intraoperative guidance and postoperative monitoring remain underexplored, suggesting that current research is more focused on design and planning rather than execution or follow-up.

Different AI architectures were applied depending on the clinical task. Detection and segmentation models, such as CNNs and U-Net variants, were commonly used to localize anatomical landmarks and classify bone density [15–17]. Prediction models, including regression approaches, LeNet-5, and LLMs, guided decisions on torque estimation, drilling protocols, and language-based planning [18, 20, 22]. Notably, the use of LeNet-5 reflected an early proof-of-concept approach rather than state-of-the-art performance, while LLMs introduced a novel paradigm by leveraging text-based knowledge instead of imaging inputs. Generative models, particularly GANs and conditional GANs, were applied to reconstruct crown morphology and occlusal details [23–28], offering enhanced surface accuracy compared with traditional CAD-based designs. Optimization frameworks such as ANN-PSO and CNN-BESO were used to design biomechanical framework [29, 30], demonstrating clear efficiency gains compared with conventional finite element approaches. For prognostic modeling, ANN, MIL, clustering, and AMDRN models were developed to predict implant survival and treatment outcomes, highlighting an emerging but less mature domain [31–34]. In summary, prediction and detection models dominated across applications, generative approaches were primarily applied to crown design, and optimization and prognostic models were less common but offered meaningful advances in framework efficiency and long-term risk assessment.

The majority of studies relied on radiographic imaging data such as CBCT or intraoral scans [15–19, 23–28, 34], while some incorporated FEA-derived simulations for biomechanics [29, 30]. More recent studies used large-scale electronic patient records [21, 31–33] or even text-based inputs through LLMs [22], reflecting an ongoing shift toward multimodal data integration. Performance was typically assessed using AUC, Dice scores, Chamfer distance, Hausdorff distance, IoU, mean deviations, or stress reduction metrics, depending on the task. Reported metrics were generally strong, with AUC values of 0.87–0.96, Dice scores around 0.75, and crown morphology deviations of 0.18–0.30 mm, all within clinically acceptable ranges.

Gold standards for validation varied but generally included expert clinician plans, technician-generated crowns, FEA benchmarks, or clinical follow-up data. Direct AI–clinician comparisons were made in at least six studies [19, 22–25, 27], showing that AI could reduce planning times by up to fourfold while maintaining comparable or superior accuracy in treatment planning and prosthesis design. However, most validations were limited to retrospective, single-center, or simulation-based datasets. Only a limited number of studies [19, 22, 31–34] incorporated real patient data or survival outcomes, and no prospective clinical trials were identified, underscoring the gap between technical feasibility and clinical adoption.

Despite promising performance, several gaps remain. Applications to esthetic anterior zones are unexplored, patient-specific adaptability in full-arch designs is lacking, and prognostic models rarely account for peri-implant disease, prosthetic complications, or patient-reported outcomes. The current outcome focus on implant survival alone contrasts with the multidimensional success criteria emphasized in clinical practice.

This review is limited by the heterogeneity of study designs and outcomes. Most models were trained on retrospective datasets of varying quality and often lacked multimodal integration. Future research should prioritize larger multicenter datasets that include systemic and patient-reported factors, consensus-based ground truths, validation across diverse clinical environments, and prospective trials. Expanding outcome measures to long-term prosthetic and patient-centered endpoints will be critical for translating AI from proof-of-concept tools to clinically reliable decision support systems.

## Conclusions

AI applications in fixed tooth- and implant-supported prosthodontics are most developed in implant site planning and crown design, with fewer studies addressing full-arch optimization or long-term prognosis. Detection and prediction models dominate, while generative and optimization models improved occlusal surface detail and framework design, and predictive models consistently support planning and risk stratification. Most models achieved high technical accuracy and efficiency gains. However, the current evidence base remains early-stage, retrospective, and simulation-focused. Future research must emphasize prospective, multimodal, and patient-centered evaluations to establish clinical reliability and broader applicability.

## Supplementary Information

Supplementary Material 1.

## Data Availability

All data generated and analyzed for the review are available upon request from the authors.

## Abbreviations

AI: Artificial intelligence
PRISMA: Preferred reporting items for systematic reviews and meta-analyses
ANN: Artificial neural network
CNNs: Convolutional neural networks
GANs: Generative adversarial networks
NNR: Neural network–based regression models
CAD/CAM: Computer-aided design/computer-aided manufacturing
CBCT: Cone-beam computed tomography
DL: Deep learning
LLMs: Large language models
DTR: Decision tree regression
FEA: Finite element analysis
PSO: Particle swarm optimization
BESO: Bi-evolutionary structural optimization
R-CNN: Convolutional neural network
