# Supplementary material for: Can artificial intelligence optimize treatment planning and outcome prediction in fixed tooth- and implant-supported prosthodontics? A scoping review
Source: BMC Oral Health. 2025 Dec 11;26:104. doi: 10.1186/s12903-025-07300-8 (PMC12809939; doi:10.1186/s12903-025-07300-8)
Supplement: Supplementary file 1 — Supplementary Material 1. [file 12903_2025_7300_MOESM1_ESM.docx]

**Additional Table 1.**

Full keyword combinations from three databases.

| **Database** | **Search strategy** |
| --- | --- |
| PubMed | ("artificial intelligence" OR "AI" OR "digital dentistry" OR "machine learning" OR "deep learning" OR "neural networks" or “supervised machine learning” OR “unsupervised machine learning”)  AND  (“prostho*” OR "fixed prosthodontics" OR "prosthetic treatment" OR “dental prosthesis” OR "prosthesis planning" OR "implant prosthodontics" OR “dental implants” OR "tooth-supported prosthesis" OR crowns)  AND  ("treatment planning" OR "decision making" OR "clinical decision support" OR "optimization") |
| Scopus | (TITLE-ABS-KEY("artificial intelligence" OR "digital dentistry" OR "AI" OR "machine learning" OR "deep learning" OR "neural networks" OR "supervised machine learning" OR "unsupervised machine learning"))  AND  (TITLE-ABS-KEY("prostho*" OR "fixed prosthodontics" OR "prosthetic treatment" OR "dental prosthesis" OR "prosthesis planning" OR "implant prosthodontics" OR "dental implants" OR "tooth-supported prosthesis" OR "crowns"))  AND  (TITLE-ABS-KEY("treatment planning" OR "decision making" OR "clinical decision support" OR "optimization")) |
| Embase | ('artificial intelligence'/exp OR 'digital dentistry'/exp OR 'machine learning'/exp OR 'deep learning'/exp OR 'neural network'/exp OR 'artificial intelligence':ti,ab,kw OR 'AI':ti,ab,kw OR 'machine learning':ti,ab,kw OR 'deep learning':ti,ab,kw OR 'neural networks':ti,ab,kw OR 'supervised machine learning':ti,ab,kw OR 'unsupervised machine learning':ti,ab,kw)  AND  ('prosthodontics'/exp OR 'dental prosthesis'/exp OR 'implant prosthesis'/exp OR 'dental implant'/exp OR 'crown'/exp OR prostho*:ti,ab,kw OR 'fixed prosthodontics':ti,ab,kw OR 'prosthetic treatment':ti,ab,kw OR 'dental prosthesis':ti,ab,kw OR 'implant prosthodontics':ti,ab,kw OR 'dental implants':ti,ab,kw OR 'tooth-supported prosthesis':ti,ab,kw OR crowns:ti,ab,kw)  AND  ('treatment planning'/exp OR 'decision making'/exp OR 'clinical decision support system'/exp OR 'optimization':ti,ab,kw OR 'treatment planning':ti,ab,kw OR 'decision making':ti,ab,kw OR 'clinical decision support':ti,ab,kw) |
